# Quality of care and suspected developmental delay among children aged 1–59 months: a cross-sectional study in 8 counties of rural China

**DOI:** 10.1186/s12887-019-1406-x

**Published:** 2019-01-31

**Authors:** Chenlu Yang, Xiaoli Liu, Yuning Yang, Xiaona Huang, Qiying Song, Yan Wang, Hong Zhou

**Affiliations:** 1 Department of Maternal and Child Health, School of Public Health, Peking University, No. 38 Xueyuan Road, Haidian District, Beijing, 100191 China; 2 United Nations International Children’s Emergency Fund China, No. 12 Sanlitun Road, Chaoyang District, Beijing, 100600 China

**Keywords:** Quality of care, Developmental delay, Children, China, Ages and stages questionnaires

## Abstract

**Background:**

The data about quality of care of more than 70 countries were available from UNICEF but little was known about China. We examined the status about quality of care and explored its associations with developmental outcomes in Chinese children.

**Methods:**

A cross-sectional study with probability proportional to size sampling method was conducted in 8 counties of rural China. A total 1927 children were assessed on development status using Ages and Stages Questionnaires-Chinese (ASQ-C) based on Chinese normative data. Nutritional status was derived from the anthropometric method following WHO guidelines. Caregivers were interviewed through household questionnaires from UNICEF’s 5th Multiple Indicator Cluster Survey to understand the quality of care, including the status of availability of children’s books, availability of playthings, support for learning, fathers’ support for learning and inadequate care. Moreover, quality of care was explored to be categorized into three levels (poor, medium and good) for overall assessment. Multivariable logistic regression model was applied to estimate the odds ratios and 95% confidence intervals between quality of care and suspected developmental delay (SDD) after adjustment for potential confounding variables.

**Results:**

The proportions of availability of children’s books, playthings, support for learning, fathers’ support for learning and inadequate care were 36.8, 91.3, 83.1, 16.4 and 4.9%, respectively. When compared to available data of more than 70 countries and areas, the quality of care in rural China was in the middle to upper level. After adjustment for potential confounding variables, multivariable analysis showed that SDD in overall ASQ remained negatively associated with availability of children’s books (odds ratio [OR] and 95% confidence interval [CI]: 1.64 [1.27–2.12]), playthings (OR and 95% CI: 2.23 [1.52–3.27]) and support for learning (OR and 95% CI: 1.81 [1.06–3.10]). When compared with children under good quality of care, children under medium and poor quality of care had higher prevalence of SDD in overall ASQ (OR and 95% CI: 1.59 [1.21–2.07]; 3.05 [1.96–4.74]).

**Conclusions:**

Quality of care in rural China still had scope for improvement. Better quality of care had negative associations with SDD.

## Background

The early years of life are a period of considerable opportunity for growth and vulnerability to harm [1]. Disadvantaged exposures and experiences in early years (prenatal to the age of 5 years) increase the risk of poor social, cognitive, and health outcomes and create a trajectory across their whole life [2]. Home environment is a primary medium for children. A mounting body of evidence suggests responsive and nurturing care play crucial roles on children’s development [3]. It is estimated that more than 250 million children under 5 years of age in low-income and middle-income countries are at risk of not attaining their developmental potential, of that number the 17.43 million that live in China [4, 5]. Of the various affecting factors, nurturing care provided by parent and family interactions is identified as an important one [4].

Based on United Nations International Children’s Emergency Fund (UNICEF), the most beneficial home settings for children’s development should be caring, safe and well-organized and children have adequate materials and opportunities to play, explore and discover [6]. UNICEF has developed specific indicators about significant aspects in the home for enhancing early childhood development, of which is quality of care, including the availability/variety of learning materials in the home, adult and paternal support for learning and school readiness, and non-adult care [6]. The data about quality of care from more than 70 countries were obtained by Multiple Indicator Cluster Surveys (MICS), Demographic and Health Surveys (DHS) and other nationally representative surveys but little was known about China [7].

Although China has rapid industrialization and economic growth in the past several decades, regional economic development disparities still remain. Researchers have concerned that children living in poor rural areas in China sometimes had few opportunities to play and learn due to resource-constrained settings and fall-behind knowledge [8, 9]. Hence, the children in rural China may get poor quality of care, which may cause poor development. Unfortunately, no study had determined the status of quality of care and explored the associations between quality of care and children’s developments in rural China.

To address these research gaps, we conducted a population-based survey in 8 counties in poor rural areas of central and western China. The aim of the present study was (a) to determine the status about quality of care and (b) to explore the associations between quality of care and developmental delay.

## Methods

### Study designs and participants

This study was a cross-sectional survey on early childhood development from October 2016 to January 2017, covering 8 rural counties in 4 central and western provinces of China (Jiangxi, Ningxia, Qinghai and Xinjiang), as part of Integrated Maternal and Child Health Development (IMCHD) project. All counties were selected by National Health and Family Planning Commission of China (NHFPC) and UNICEF due to their poor socio-economic development. A multistage sampling method was employed in this survey. First, 15 administrative villages per county were selected at random with population proportional to size (PPS) method. PPS method is a sampling procedure under which the probability of a unit being selected is proportional to the size of the ultimate unit, giving larger clusters a greater probability of selection and smaller clusters a lower probability [10]. Next, 2 groups per administrative village were selected at random with PPS method. Groups are the basic units of daily life and spontaneously and naturally existing within rural areas in China. Within each selected groups, simple random sampling was used to select 8 households with at least 1 child aged under 60 months, according to the full registration lists provided by local village doctors. Children who were under 60 months, lived locally more than 6 months, and accessed medical services locally were included in our investigation. Children with severe physical disability or critical illness (impairment of vision, hearing, walking, etc.) were excluded. The primary caregiver of child was respondent during the face-to-face investigation. For left-behind children (defined as those with one or both parents who had left home to work elsewhere), another parent left behind, children’s grandparents or other relatives answered the questionnaires. Interviews with caregivers and children were conducted by UNICEF, Peking University, Lanzhou University, Capital Medical University staff working with local health workers. The household questionnaires were developed from 5th Multiple Indicator Cluster Surveys (MICS5) of UNICEF [11].

### Key study variables

#### Quality of care

According to UNICEF [6], five indicators were employed to assess quality of care and the definitions were as follows:

Availability of children’s books: child aged 1–59 months had three or more children’s books.

Availability of playthings: child aged 1–59 months played with two or more types of playthings.

Support for learning: as any household members age 15 or over engaged in four or more of following activities with child aged 36–59 months in last 3 days: a) read books to or looked at pictures books with the child; b) told stories to the child; c) sang songs to or with the child, including lullabies; d) took the child outside the home, compound, yard and enclosure; e) played with the child; f) named, counted, or drew things to or with the child.

Father’s support for learning: child’s father engaged in four or more above-mentioned activities with child aged 36–59 months in last 3 days.

Inadequate care: child aged 1–59 months was left alone or in the care of another child younger than 10 years for more than one hour at least once in the last week.

In this report, for children aged 1–35 months, 3 indicators were employed to categorize the quality of care: “availability of children’s books”, “availability of playthings” and “without inadequate care”. Good quality of care was defined as meeting 3 items; medium quality of care was defined as meeting 2; poor quality of care was defined as meeting 1 or 0. For children aged 36–59 months, 4 indicators were employed to categorize the quality of care: “availability of children’s books”, “availability of playthings”, “support for learning” and “without inadequate care”. Good quality of care was defined as meeting 4 items; medium quality of care was defined as meeting 2 or 3; poor quality of care was defined as meeting 1 or 0.

#### Malnutrition

Children were measured bareheaded and barefoot for body length/height and weight by two interviewers in each group sampled. Using the Length Meter with Model SH-8093 Horizontal Type for children aged 1 to 23 months (Suhong Weighing Apparatus Factory, Hengshui, China) and the Height Meter with Model SZ-200/120 Type for children aged 24 to 59 months (Wujin Weighing Apparatus Factory, Changzhou, China), each child’s length/height was measured to the nearest 0.1 cm. A scale was used to measure weight to the nearest 0.05 kg (OMRON electronic scale HN-289-BK; OMRON healthcare, Dalian, China). Each measurement was performed twice and the average value was used for analysis. Length/height-for-age Z-scores (HAZ), weight-for-age Z-scores (WAZ) and Weight-for- length/height Z-scores (WHZ) were computed based on WHO 2006 Child Growth Standard [12]. HAZ < − 2 was defined as stunting; WAZ < − 2 was defined as underweight; WHZ was defined as wasting. Any one or more the three conditions, stunting, underweight or wasting, was defined as malnutrition.

#### Suspected developmental delay

The Ages and Stages Questionnaires (ASQ), a Parent-Completed Child-Monitoring System, is an accurate, cost-effective and parent-friendly way to identify children with potential developmental problems [13]. The Ages and Stages Questionnaires-Chinese (ASQ-C) is the Chinese version of Ages and Stages Questionnaires-third edition (ASQ-3), which has been found to be a validated developmental screening instrument for Chinese children [14]. The ASQ-C consists of 21 questionnaires and different child’s age group has corresponding one. The corrected age was used for preterm (defined as gestational age under 37 weeks) who was less than 2 years old during the investigation to select questionnaires, according to the official guideline of ASQ-3 [13]. Each questionnaire in the ASQ-C consists of 30 items covering five domains: communication (CM), gross motor (GM), fine motor (FM), problem solving (CG) and personal-social (PS). The answer of each item ‘yes’ is scored 10 points, ‘sometimes’ is scored 5 points and ‘not yet’ is scored 0 points. The sum scores of every domain were compared with the national normative cut-off point of China. ASQ only can be used for children aged more than 1 month, so children aged 1–59 months and their primary caregivers were included in this report. Children whose scores were lower than the cut-off point of China in any domain were regarded as suspected developmental delay (SDD).

#### Covariates

The questionnaire also included questions on the age, gender, gestational age, birthweight, birth order of the children and whether children were left-behind (defined as those with one or both parents who had left home to work elsewhere) or not and on the socio-economic characteristics of the household (income and education of the primary caregivers). In our report, household net income was equal to total household income for the last year minus the production income (produced gain, poultry being sold, etc.), income from working, and government funding. Household expenses included agricultural productive expenses (seeds, fertilizers, pesticides, feed, etc.), living expenses (clothing, food, household appliance, etc.), health care expenses, and tax [15, 16]. The annual net income of household divided by the total population of the family made per capita net income of household. The families were categorized into five classifications (poorest/poor/middle/richer/richest) based on the quintiles in the distribution of household per capita income in surveyed areas. In our study, all information about family income were provided by our interviewees.

### Statistical analysis

The data was presented as frequencies and percentage. Chi-square tests were used to access quality of care by gender. Trend chi-square tests were used to access quality of care by socioeconomic classifications and age groups. In order to determine the association between quality of care and SDD, we conducted Chi-square tests, Trend chi-square tests and multivariable logistic regression analyses, with SDD as the dependent variable. The effects of potential confounders in our analyses were child gender, child age, preterm, birth weight, child order, left-behind child, malnutrition, caregiver, socioeconomic classification and caregiver’s education. The data was analyzed by using Statistical Package for the Social Sciences (SPSS) 19.0 software package and a *p*-value (2-tailed) less than 0.05 was considered statistically significant.

## Results

### Basic characteristics of the subjects

A total of 1927 children and their primary caregivers were recruited. As shown in Table 1, 53.9% of the children were boys and 49.9% were aged 12–35 months. The proportion of preterm was 4.9%. The proportion of low birth weight infants and macrosomia were 5.6 and 5.3%, respectively. Almost 40% of the children were the first child for their parents, 46.1% were left-behind children and 6.3% were malnourished. Most of the caregivers were mothers (66.1%), while 8.5% were fathers.

**Table 1:** Basic characteristics of children and caregivers in the study

Characteristic	*n*	%
Gender		
Male	1038	53.9
Female	889	46.1
Age(months)		
1–11	369	19.1
12–35	962	49.9
36–59	596	30.9
Preterm^a^		
Yes	87	4.9
No	1705	95.1
Birthweight^b^		
Low(< 2500 g)	102	5.6
Good(2500- < 4000 g)	1632	89.1
Macrosomia(> = 4000 g)	97	5.3
Child order		
1	757	39.3
> =2	1170	60.7
Left-behind child		
Yes	889	46.1
No	1038	53.9
Malnutrition^c^		
Yes	119	6.3
No	1778	93.7
Caregivers		
Mother	1274	66.1
Father	163	8.5
Grandmother	348	18.1
Grandfather	122	6.3
Other relatives	20	1.0
Socioeconomic classification^d^		
Poorest	422	22.6
Poor	323	17.3
Middle	412	22.1
Richer	241	12.9
Richest	469	25.1
Caregiver’s education		
Illiteracy	249	12.9
Primary school	458	23.8
Middle school	855	44.4
High school	220	11.4
College degree or above	145	7.5

^a^135 caregivers without this information

^b^96 caregivers without this information

^c^30 children without this information

^d^60 caregivers without this information

Of remaining caregivers, 18.1% were grandmothers, 6.3% were grandfathers and 1.0% were other relatives (older sisters/brothers, aunts, uncles, etc.). 44.4% of the caregivers had middle education, but the proportion of illiteracy was as high as 12.9% and only 7.5% were well educated with a college or above education level.

### Quality of care

As shown in Table 2, only one third of the children had access to children’s books (36.8%) and the majority of the children had access to playthings (91.3%). More than 80% of the children got support for learning but only 16.4% of the children got father’s support for learning. The proportion of inadequate care was 4.9%. For overall assessment, the proportion of children under poor quality of care was as high as 9.2%. Difference based on gender was not statistically significant for all items (Table 2).

**Table 2:** The status of quality of care and comparison of different status of quality of care by gender

	*N* ^a^	*n* (%)	Male *n* (%)	Female *n* (%)	*P*
Five items					
Availability of children’s books	1927	710 (36.8)	377 (36.3)	333 (37.5)	0.606
Availability of playthings	1927	1759 (91.3)	950 (91.5)	809 (91.0)	0.686
Support for learning	596	495 (83.1)	274 (81.3)	221 (85.3)	0.194
Father’s support for learning	596	98 (16.4)	57 (16.9)	41 (15.8)	0.723
Inadequate care	1916	93 (4.9)	44 (4.3)	49 (5.5)	0.201
Overall assessment					
Poor quality of care	1916	176 (9.2)	96 (9.3)	80 (9.0)	0.943
Medium quality of care	1916	1111 (58.0)	599 (58.2)	512 (57.8)	
Good quality of care	1916	629 (32.8)	335 (32.5)	294 (33.2)	

^a^in accordance to MICS5 definitions, “availability of children’s books”, “availability of playthings” and “inadequate care” are applicable for children aged 1–59 months (*N* = 1927). “Support for learning” and “father’s support for learning” are applicable for children aged 36–59 months (*N* = 596). 11 caregivers had forgot the details about inadequate care (*N* = 1916)

**Fig. 1:**
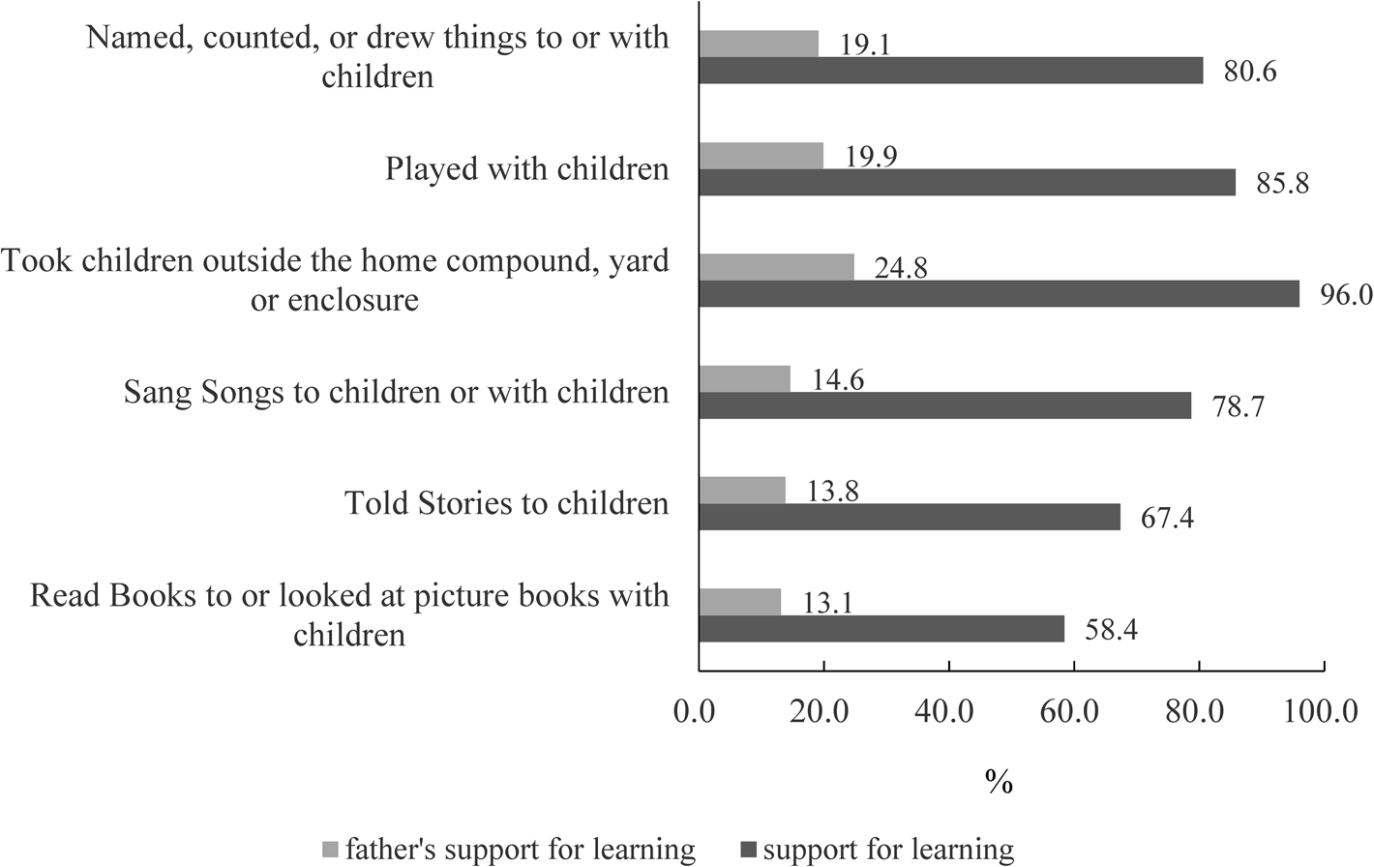
Different kinds of support for learning among children aged 36–59 months

**Fig. 2:**
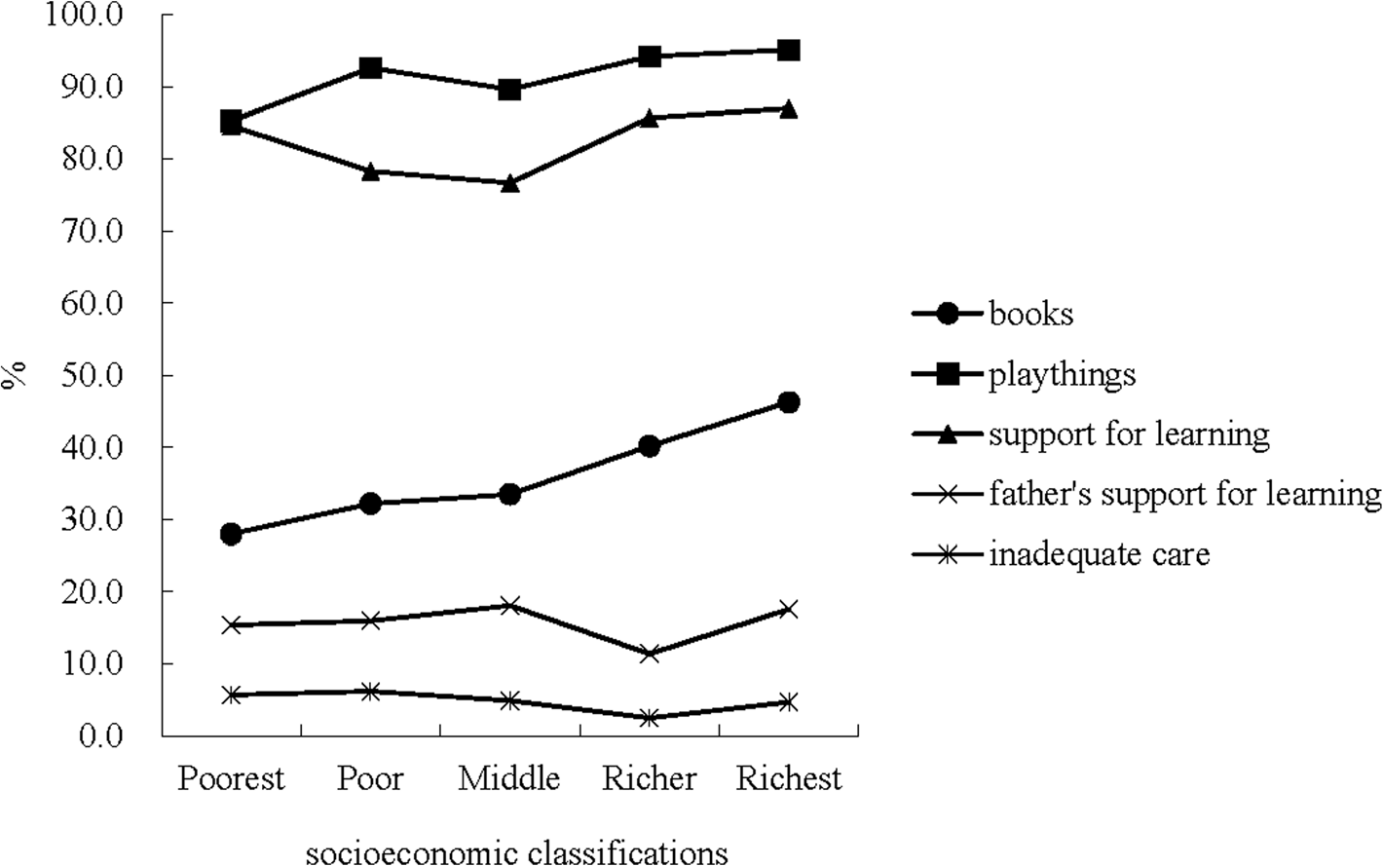
Comparison of different status of quality of care by socioeconomic status

**Fig. 3:**
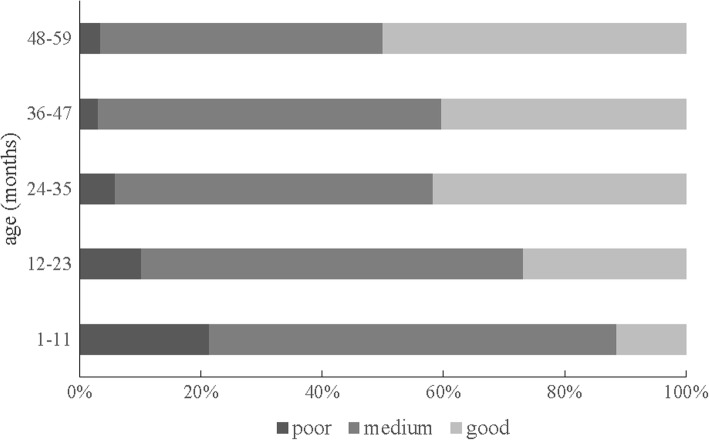
Comparison of different status of quality of care by age groups

For children aged 36–59 months, taking children outside the home, compound, yard and enclosure was the most popular activity of support for learning and father’s support for learning (96.0 and 24.8%); reading books to or looking at pictures books with the children was the lowest one (58.4 and 13.1%) (Fig. 1). Figure 2 presents the proportions of different status of quality of care by socioeconomic classifications. Availability of children’s books and playthings, increased by the increasing socioeconomic level by using trend chi-square tests (*P* < 0.001; *P* < 0.001). For overall assessment, trend chi-square tests showed good quality of care increased with age growth (*P* < 0.001) (Fig. 3).

### Associations between quality of care and SDD

**Fig. 4:**
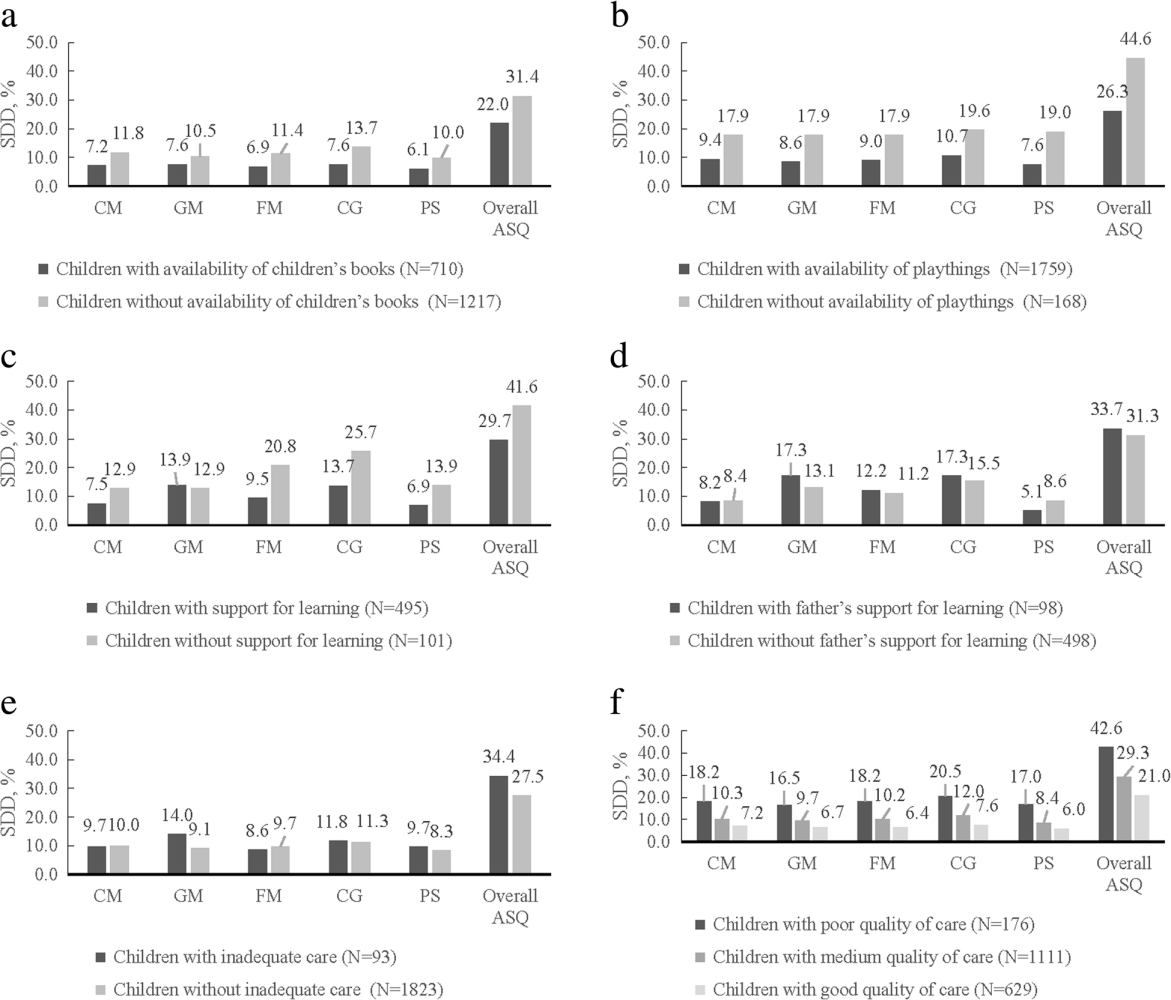
Comparison of prevalence of SDD among children under different status of quality of care. **a** Comparison of prevalence of SDD among children with/without availability of children’s books. **b** Comparison of prevalence of SDD among children with/without availability of playthings. **c** Comparison of prevalence of SDD among children with/without support for learning. **d** Comparison of prevalence of SDD among children with/without father’s support for learning. **e** Comparison of prevalence of SDD among children with/without inadequate care. **f** Comparison of prevalence of SDD among children with poor/medium/good quality of care

Figure 4 showed the comparison of prevalence of SDD among children under different status of quality of care. Children with availability of children’s books and playthings had lower prevalence of SDD in any domain of ASQ and overall ASQ (*P* < 0.05) (Fig. 4a, b). Support for learning had negative associations with SDD in FM, CG, PS and overall ASQ (*P* < 0.05) (Fig. 4c). Trend chi-square tests showed children with better quality of care had lower prevalence of SDD in any domain of ASQ and overall ASQ (*P* < 0.001) (Fig. 4f).

Multivariable (adjusted) regression analysis between quality of care and SDD in ASQ were reported in Table 3. After adjustment for all variables in Table 1, SDD in CM, GM, FM, CG and overall ASQ were negatively associated with availability of children’s books (*P* < 0.05). SDD in any domain and overall ASQ still remained negatively associated with availability of playthings (*P* < 0.05). Support for learning had negative associations with SDD in FM, CG and overall ASQ (*P* < 0.05). When compared with children under good quality of care, it was observed that children under medium quality of care had higher prevalence of SDD in GM, FM, CG and overall ASQ and children under poor quality of care had higher prevalence of SDD in any domain of ASQ and overall ASQ.

**Table 3:** Multivariable logistic regression analysis between quality of care and SDD in ASQ

			SDD in CM	SDD in GM	SDD in FM	SDD in CG	SDD in PS	SDD in overall ASQ
			Adjusted OR (95%CI)^a^	Adjusted OR (95%CI)^a^	Adjusted OR (95%CI)^a^	Adjusted OR (95%CI)^a^	Adjusted OR (95%CI)^a^	Adjusted OR (95%CI)^a^
Five items	Availability of children’s books	No vs. yes	**1.56(1.04–2.34)**	**1.58(1.06–2.36)**	**1.68(1.13–2.51)**	**1.93(1.33–2.81)**	1.50(0.98–2.29)	**1.64(1.27–2.12)**
	Availability of playthings	No vs. yes	**1.92(1.12–3.30)**	**2.28(1.36–3.81)**	**1.95(1.17–3.25)**	**2.16(1.33–3.51)**	**2.64(1.52–4.58)**	**2.23(1.52–3.27)**
	Support for learning	No vs. yes	1.75(0.73–4.22)	0.98(0.45–2.15)	**2.96(1.50–5.83)**	**3.13(1.68–5.83)**	2.25(0.94–5.36)	**1.81(1.06–3.10)**
	Father’s support for learning	No vs. yes	0.53(0.21–1.33)	0.62(0.31–1.24)	0.72(0.34–1.54)	0.74(0.39–1.40)	1.67(0.52–5.34)	0.64(0.38–1.09)
	Inadequate care	No vs. yes	1.27(0.52–3.09)	0.67(0.33–1.38)	1.29(0.56–2.99)	1.21(0.57–2.55)	0.90(0.37–2.18)	0.93(0.55–1.57)
Overall assessment	Quality of care	Poor vs. good	**2.45(1.30–4.61)**	**3.97(2.09–7.53)**	**3.32(1.77–6.22)**	**3.85(2.15–6.90)**	**2.80(1.44–5.45)**	**3.05(1.96–4.74)**
		Medium vs. good	1.44(0.94–2.20)	**1.71(1.11–2.63)**	**1.72(1.12–2.65)**	**1.58(1.07–2.34)**	1.28(0.82–1.99)	**1.59(1.21–2.07)**

^a^adjustment for all variables in Table 1; bold indicates statistical significant (*P* < 0.05)

No statistically significant differences were observed between father’s support for learning and inadequate care and SDD both before and after adjustments.

## Discussion

In this report, we reported quality of care in surveyed areas in China; we observed socioeconomic classifications were associated with availability of children’s books and playthings and age were associated with quality of care; we found that availability of children’s books, playthings and support for learning had negative associations with SDD and better quality of care was a protective factor for SDD.

### Quality of care

Quality of care is one of the crucial areas about measuring early childhood development. Overall, when compared to available data of UNICEF (Last update: November 2017) [7], the quality of care in our surveyed areas was in the middle to upper level, but it still had scope for improvement. For example, the proportion of availability of children’s books in Belarus was as high as 92.0%, while it was only 36.8% in our surveyed areas. Additionally, father’s support for learning was as low as 16.4% in our surveyed areas, which had gaps with many countries (84.9% in Qatar, for instance).

In the field of public health, development of effective intervention strategies requires an understanding of high-risk populations. We compared the different status of quality of care by gender and socioeconomic classification, which can help to identify vulnerable groups. Gender, as an important demographic characteristic, may play a role in quality of care. For example, a previous study has reported family members show more preference to, give attention to, talk to and interact more with boys than girls in Ethiopia or other African countries [17]. Traditional concept of Chinese child-rearing behaviors was “son preference”, which meant caregivers tended to give boys preferential treatments than girls. In our study, we found that gender had no impact on the quality of care, and girls got equal opportunities to learn, play and develop. Researchers have revealed that poverty is associated with a mass of health problems of children, parental stress and strains in parent–child relationships [18–20]. For example, extreme poverty was strongly linked to restricted learning opportunities and inadequate stimulation at home [17]. We observed positive associations between socioeconomic classifications and availability of children’s books and playthings. Therefore, it might be suggested that future intervention could focus on the poor children.

The most common way of support for learning in our surveyed areas was taking children outside, and the rates of telling stories and reading books were at a relatively low level. The possible explanation was that caregivers (e.g., elder ones and illiterate ones) lacked the perceptions and skills of telling stories and reading books. In this context, caregivers would be at the core of the intervention. Future intervention programme should highlight the significance and skills about early child development to caregivers and help them to overcome obstacles. Health promotion and education should be conducted, which can help caregivers to do better use of books and playthings, teach them how to read books, tell stories and play with children. For example, researchers used a counseling card (the Mother’s Card) for promotion effective play and communication between caregivers and children in China and it was proved helpful and effective [21].

In addition, we found the proportion of father’s support for learning was quite low. Previous studies have reported mothers and fathers appeared to engage in different types of interaction with their child and produce different outcomes [22–24]. However, “Absent fathers”, especially in low-income families, has been a concern in many fields, such as social and behavioral science departments and governments [25]. Traditional concept of Chinese families was that men played the key role in the society (taking financial responsibility for family members, for instance) while women played the key role in the family (taking nursing responsibility, for instance) and it was common that grandparents helped young couples to bring up children. Researchers have found that fathers’ involvement in parenting was less than mothers’ in Chinese families [26]. Although father’s participation in child-rearing has been highlighted, fathers continue to spend less time with their children than do mothers [23]. Hence, the limited father’s participation in child-rearing needed improvements.

We found about one tenth of children got poor quality of care, and we observed positive associations between age growth and good quality of care, which indicated younger children needed more attention. As reported by another study in Iran, children aged 18–30 months got more opportunities in average book reading, storytelling, and singing duration than children under 17 months [27]. Additionally, other researchers observed the youngest group faced the most serious deprivation of learning resources, which could be result from an inaccurate belief in rural China that infants knew nothing except eating and sleeping [9]. In our study, we obtained similar results and we supported the younger children needed more attention as a vulnerable group.

### Associations between quality of care and SDD

We found that availability of children’s books, playthings, support for learning and better quality of care were protective factors for SDD, which was consistent with previous studies. For example, a birth cohort in Brazilian has revealed that children who have not been told stories in the previous week and children who did not have children’s literature at home were more likely to show suspected developmental delay [28]. Reading aloud and provision of toys are associated with better child cognitive and language development at 21 months among low-income Latino children [29]. A pregnancy cohort has highlighted that strategies that assist parents with infant interaction skills are protective factors for children at risk of developmental delay [30]. Our multivariable analysis confirm these findings and improving quality of care is a feasible and effective way to enhance child development.

Researchers have highlighted that fathers’ positive parenting produced better cognitive, social, and emotional development of children [31]. Positive psychological and emotional aspects of father participation in child-rearing may prevent children from developing symptoms of depression in their pre-teen years [32]. However, we found father’s support for learning was not statistically associated with SDD in surveyed areas. This may partly because limited father participation was insufficient to show positive child development outcomes. Another possible explanation may be that other relatives (grandfathers, older brothers, for instance) offered “father’s support for learning”, resulting in a bias for analysis. Although no difference was observed between children with/without father’s support for learning, father’s involvements warranted consideration in child health and development, especially in Chinese cultural context. Based on many studies about father-child relationships, a significant contribution of a father to child’s whole life was reported [33–35].

Although we didn’t figure out statistical significance between inadequate care and SDD, inadequate care was always dangerous for young children and may cause accidental injuries.

### Strengths and limitations

Child development comes to be a global issue and its significance is highlighted by a body of studies [3, 4, 36]. However, gaps still exist in China, especially in poor areas. There is a scarcity of literature in rural China regarding the state of child development for children under 60 months that go beyond nutrition and growth outcomes. Our study obtained the developmental outcomes among Chinese rural children by Chinese national cut-off of ASQ-C. Moreover, the indicators about quality of care of MICS have been used among many countries and areas, but there is a lack of information among Chinese children. To our best knowledge, our study was the first report that assessed the status of qualify of care and determined its contributions to SDD in rural China. Our findings may help to recognize vulnerable groups and confirm the associations between quality of care and SDD, which may contribute to inform invention projects about improving child development in rural China.

The present study was subject to certain limitations. First, our data were cross-sectional. Although we demonstrated significant impacts of quality of care on SDD, causal and temporal associations could not be inferred. Additional longitudinal studies, in which biological and family and environmental factors during pregnancy and the postpartum period can be prospectively measured, should be conducted to confirm our findings. Another limitation was that ASQ is only a screen tool for developmental delay. The potential bias caused by misclassification error should be considered when interpreting the findings.

## Conclusions

To conclude, our study reported the status of quality of care in poor rural areas of central and western China, and provided evidence about associations between quality of care and SDD. Our findings highlighted the importance of quality of care among children in rural areas of China, and can be used for identification the children at high risk of developmental delay and for future intervention programme.

## Abbreviations

ASQ: Ages and Stages Questionnaires
CG: Problem solving
CM: Communication
DHS: Demographic and Health Surveys
FM: Fine motor
GM: Gross motor
HAZ: Length/height-for-age Z-scores
IMCHD: Integrated Maternal and Child Health Development
MICS: Multiple Indicator Cluster Surveys
NHFPC: National Health and Family Planning Commission of China
PPS: Population proportional to size
PS: Personal-social
SDD: Suspected developmental delay
UNICEF: United Nations International Children’s Emergency Fund
WAZ: Weight-for-age Z-scores
WHZ: Weight-for- length/height Z-scores
